# Recent Advances in Psychopharmacology

**DOI:** 10.3390/ph18111602

**Published:** 2025-10-23

**Authors:** Roos van Westrhenen, Allan H. Young

**Affiliations:** 1 Parnassia Group, Outpatient Clinic Pharmacogenetics, Overschiestraat 57, 1062 HN Amsterdam, The Netherlands; 2 Department of Psychological Medicine, Institute of Psychiatry Psychology and Neuroscience, King’s College London, London SE5 8AG, UK; 3 Department of Psychiatry, St. John’s National Academy of Health Sciences, Bangalore 560034, Karnataka, India; 4 Division of Psychiatry, Imperial College London, London SW7 2AZ, UK

Delivering clinical trials is a challenge, most notably due to recruitment, especially among individuals with psychiatric disorders. Questions repeatedly arise regarding the most reliable and tractable outcome measures. Such factors may have influenced the cessation of psychopharmacological discovery among drug companies such as Astra Zeneca, Pfizer, Glaxo Smith Kline and Eli Lilly [1]. Still, in a 2023 overview of all recent and current phase 2 or phase 3 clinical trials, the authors discovered *n* = 43 trials on schizophrenia medication, *n* = 11 on bipolar disorder, *n* = 56 on major depressive disorder, *n* = 29 on anxiety disorder and trauma-related disorders, and *n* = 17 on medication for the treatment of substance disorders [2]. One must bear in mind that, in general, the availability of new psychopharmacological treatments to patients takes nearly nine years. The likelihood of eventual drug approval in psychiatry is only 6.2%, and not all approved treatments are funded by local health care authorities. Thus, novel strategies in psychiatry are urgently needed but difficult to deliver [3].

Enhancing medication prescription with pharmacogenetics is another important strategy. In general, only one-third of patients respond well to and tolerate treatment with available medication in psychiatry [4,5]. Today, medication selection in psychiatry relies on a trial-and-error approach based mainly on physicians’ experience [4,5]. Pharmacogenetic testing can help in this process by determining the person-specific genetic factors that may predict clinical response and side effects associated with genetic variants that impact drug-metabolizing enzymes, drug transporters, or drug targets [4,5,6,7].

This Special Issue of *Pharmaceuticals* features a curated collection of original research articles and reviews that showcases the recent advances in psychopharmacology. For a detailed list of contributions, please refer to the back matter.

In this issue, a new overview (Bozzatello et al.) is provided on the molecules approved by the main drug agencies, the Food and Drug Administration (FDA) and the European Medicines Agency (EMA), from 2018 to date, along with new indications and new formulations of existing medications for schizophrenia, bipolar disorder, major depressive disorder, and anxiety disorders. It was concluded that the available literature on new advances in pharmacotherapy for psychiatric disorders suggests that the direction of research is to identify new molecules or new formulations of existing drugs to personalize treatments as much as possible while minimizing adverse effects, taking into account pharmacogenetic and digital phenotyping approaches.

Another review (Donello et al.) describes the antidepressive action of a novel class of neuroplastogens that are modulators of the NMDA Receptor (NMDAR), including not only ketamine but also the stinels—rapastinel, apimostinel, and zelquistinel.

Comai et al. hypothesize that neural plasticity is epigenetically regulated by precise Ca^2+^ quanta entering cells via NMDARs. Stimuli reach receptor cells (specialized cells that detect specific types of stimuli and convert them into electrical signals) and change their membrane potential, regulating glutamate release in the synaptic cleft. Free glutamate binds ionotropic glutamatergic receptors regulating NMDAR-mediated Ca^2+^ influx. Quanta of Ca^2+^ via NMDARs activate enzymatic pathways, epigenetically regulating synaptic protein homeostasis and synaptic receptor expression; thereby, Ca^2+^ quanta, via NMDARs, control the balance between long-term potentiation and long-term depression.

Thereafter, an exciting example from newly registered medications in psychopharmacology is described by de Freitas Menezes Sobreiro et al.: a preliminary report on esketamine nasal spray as an effective agent in therapy-resistant depression.

Varney et al. describe a study exploring pharmacogenetics in psychosis patients as well as in mood disorder patients in the UK Biobank cohort. With a diverse sample of 453 participants, the influence of *CYP1A2*, *CYP2D6*, and *CYP3A4* variation on three antipsychotic treatment outcomes was investigated: participant-reported adverse antipsychotic drug reactions, health-related quality of life, and the dose of antipsychotic medication prescribed. Over half of the sample (62.9%) were carriers of an allele associated with altered metabolism of antipsychotic medications on *CYP2D6* or *CYP3A4*, the two genes with pharmacogenetic guidelines for antipsychotic medications. Ultrarapid CYP2D6 metabolisers reported significantly lower levels of adverse antipsychotic drug reactions than normal CYP2D6 metabolisers. This suggests that even when looking at a small number of cytochrome P450 genes, carrying an allele associated with altered antipsychotic medication metabolism is relatively common, and the CYP genotype influences antipsychotic treatment outcomes.

A second analysis of UK Biobank data by a different group (Wong et al.) investigated the effects of CYP2C19 metaboliser phenotypes on several clinical outcomes derived from primary care records, including multiple measures of antidepressant switching, discontinuation, duration, and side effects. In this dataset, 24,729 individuals were prescribed citalopram, 3012 individuals were prescribed escitalopram, and 12,544 individuals were prescribed sertraline. CYP2C19-poor metabolisers on escitalopram were more likely to switch antidepressants, have side effects following first prescription, and be on escitalopram for a shorter duration compared to normal metabolisers. CYP2C19-poor and -intermediate metabolisers on citalopram also exhibited increased odds of discontinuation and shorter durations relative to normal metabolisers.

The contribution by Barabassy et al. proceeds with a review of 30 studies on the transdiagnostic efficacy of Cariprazine, showing therapeutic benefits on positive, negative, manic and depressive symptoms. Preliminary positive effects were seen on anxiety, hostility, and cognitive symptoms across disorders.

In a review by Rosian et al. on the role of vitamin D in depression, a search yielded 70 articles that were assessed, and an inverse relationship emerged between serum 25(OH)D levels and depression, highlighting beneficial effects of vitamin D supplementation on depressive symptoms. Therefore, the authors suggest that vitamin D could represent an adjunctive therapy in the management of MDD.

Sousa Silva Bonasser et al. reviewed CYP2C19 variants across populations with depression and emphasized the need to clarify the role of different CYP2C19 variants in major depressive disorder to optimize treatment strategies.

To conclude, given the fact that psychiatric disorders are complex and often highly influenced by many factors, it seems crucial that we work from all relevant angles to find improvements to the available treatment strategies, add new ones, keep refining our assessments of participants in clinical trials, and, at the same time, keep investigating the underlying pathological processes of psychiatric disorders and symptoms.
